# Naïve helper T cells with high CD5 expression have increased calcium signaling

**DOI:** 10.1371/journal.pone.0178799

**Published:** 2017-05-31

**Authors:** Claudia M. Tellez Freitas, Garrett J. Hamblin, Carlee M. Raymond, K. Scott Weber

**Affiliations:** Department of Microbiology & Molecular Biology, Brigham Young University, Provo, Utah, United States of America; University of Iowa, UNITED STATES

## Abstract

The adaptive immune response is orchestrated by T helper cells and their function is dependent upon interactions between the T cell receptor (TCR), peptide MHC (pMHC) and co-receptors. TCR-pMHC interactions initiate calcium signaling cascades which determine T cell activation, survival, proliferation and differentiation. CD5 is a co-receptor that plays an important role in regulating T cell signaling and fate during thymocyte education. CD5 surface expression on mature single positive thymocytes correlates with the TCR signal strength for positive selecting self-ligands. CD5 also plays a role in T cell function after thymic development is complete. Peripheral T cells with higher CD5 expression respond better to foreign antigen than those with lower CD5 expression and CD5-high T cells are enriched in memory populations. In our study, we examined the role of CD5 expression and calcium signaling in the primary response of T cells using two *Listeria monocytogenes* specific T helper cells (LLO118 and LLO56). These T cells recognize the same immunodominant epitope (LLO_190-205_) of *L*. *monocytogenes* and have divergent primary and secondary responses and different levels of CD5 expression. We found that each T cell has unique calcium mobilization in response to *in vitro* stimulation with LLO_190-205_ and that CD5 expression levels in these cells changed over time following stimulation. LLO56 naïve T helper cells, which expresses higher levels of CD5, have higher calcium mobilization than naïve LLO118 T cells. Three days after *in vitro* stimulation, LLO118 T cells had more robust calcium mobilization than LLO56 and there were no differences in calcium mobilization 8 days after *in vitro* stimulation. To further evaluate the role of CD5, we measured calcium signaling in CD5 knockout LLO118 and LLO56 T cells at these three time points and found that CD5 plays a significant role in promoting the calcium signaling of naïve CD5-high LLO56 T cells.

## Introduction

Helper T cells play a critical role in adaptive immunity by orchestrating and regulating the immune response [1, 2]. In large part, the binding properties of the T cell receptor (TCR) regulates the development, activation, and proliferative response of T lymphocytes [3, 4]. In the thymus, T cells are selected according to their avidity for self-peptide/MHC complexes. The TCR must be able to recognize self-peptide/MHC complexes with enough affinity to transduce a signal during positive selection while not binding so tightly that they are negatively selected [4–6]. TCR avidity and signal strength plays a key role in T cell function (calcium signaling, cytokine production, T cell proliferation and differentiation) [7–9]. In addition to the TCR and its interaction with peptide MHC (pMHC), multiple receptors such as CD4, CD8, PD-1, and CTLA-4 play a key role in determining whether TCR:pMHC binding results in T cell activation or anergy. CD5 is known to be a negative regulator of TCR signaling in developing thymocytes and its expression level in naïve T cells is determined during thymic development. CD5 levels are set during positive selection according to the strength of the TCR-self-peptide/MHC interaction. Typically, the stronger the avidity for self-peptide/MHC the higher the CD5 surface expression [10–13]. After completing thymic development, T cells with higher CD5 expression respond better to foreign antigen than those with lower CD5 expression and CD5-high T cells are enriched in memory populations [14, 15]. Although there are studies examining the role of T cell CD5 expression during thymic development and CD5-high cells are enriched in memory cell populations, it is not clear how CD5 is involved in calcium signaling during a helper T cell primary response. To better understand the role of CD5 in a T cell primary response to foreign antigen, we examined the *in vitro* calcium responses of CD5-high and CD5-low T helper cells that respond to the same epitope of *Listeria monocytogenes*.

Calcium (Ca^2+^) is a ubiquitous second messenger important for a wide range of cellular functions. Ca^2+^ signaling plays an important role in T cell activation, cytokine production, proliferation and cell fate and is determined by TCR interactions with the pMHC complex as well as additional co-receptors [9, 16]. Ca^2+^ signaling has been well characterized in lymphocytes and the calcium signal for specific helper T cell subsets has been identified, suggesting a strong relationship between Ca^2+^ mobilization in T helper cells and their functional response [17, 18]. TCR engagement with pMHC initiates signal transduction pathways that result in a dramatic increase of intracellular Ca^2+^ [19, 20]. Increases in intracellular Ca^2+^ enables transcription factors to enter the nucleus and turn on genes that play a critical role in immune responses. For example, NFAT, NF-κB, AP-1, and the Oct family transcription factors initiate transcription of the interleukin-2 (IL-2) gene [21]. IL-2 production is important for T cell proliferation and survival and plays a key role in promoting effector and memory cell differentiation [22–24]. Thus, TCR-dependent Ca^2+^ signals are essential for robust T cell primary and secondary immune responses.

The TCR avidity for self-peptide/MHC complex during selection affects the function and maintenance of these cells in the periphery and how they respond to infection [25, 26]. CD5 is a monomeric cell surface glycoprotein expressed on thymocytes, mature T cells, and a subset of B cells. High TCR avidity for self-peptide/MHC results in high surface expression of CD5 on double positive and single positive thymocytes, whereas lower avidity is correlated with lower surface expression of CD5 [10]. CD5 has been shown to negatively regulate the TCR signal during thymic development [27]. CD5 expression and Ca^2+^ mobilization correlate with TCR signal strength and T cell fate [3, 5, 28]. However, mature naïve T cells with higher expression levels of CD5 appear to respond better to foreign ligands, suggesting that CD5 influences T cell responsiveness at the post-selection level [14, 16, 29]. Thus, it appears that the negative regulatory effect of CD5 in the thymus may not depend on the extracellular region of CD5 whereas the positive co-stimulatory effect of CD5 in the periphery is dependent on extracellular engagement of an endogenous ligand (CD5 or CD5L) [30–32]. While the exact function of these CD5 ligands is unclear, there is evidence that CD5L (CD72; a C-type ligand) binds to CD5 and that CD5 is homophillic and may bind to CD5 on other cells [33]. Thus, CD5 has a critical and divergent role in regulating T cell activation depending on the time and location of activation.

LLO56 and LLO118 are two T helper cells that recognize the same immunodominant epitope (LLO_190-205_) of *L*. *monocytogenes* and have divergent primary and secondary responses. They differ by 15 amino acids in their TCR sequences and have unique responses to *L*. *monocytogenes* infection *in vivo*, LLO118 has a better primary response whereas LLO56 has a more robust secondary response [25]. Previous analysis of thymocytes and T cells revealed that LLO56 has higher levels of CD5 and a more robust IL-2 response in addition to a reduced primary response caused by increased cell death compared to LLO118 T cells [25, 26]. In order to better understand how CD5 levels affect T cell activation in cells that have completed thymic development, we determined to evaluate calcium signaling in LLO56 (CD5-high) and LLO118 (CD5-low) T cells. We also measured calcium signaling in CD5 knockout LLO118 and LLO56 T cells to better elucidate the role of CD5 in calcium signaling after thymic development is finished. This was accomplished by measuring LLO118 and LLO56 calcium mobilization at three different time points during the T cell response: Naïve T helper cells, day 3 post-stimulation, and day 8 post-stimulation. We also measured calcium signaling in CD5 knockout LLO118 and LLO56 T cells at these time points and found CD5 plays a significant role in promoting the calcium signaling of naïve CD5-high T cells, but does not alter calcium mobilization levels at later time points.

## Materials and methods

### Mice

LLO56 (B6 Thy-1.1^+^ Rag1^−/−^), LLO118 (B6 Ly5.1^+^ Rag1^−/−^) and CD5 knockout (KO) LLO56 and LLO118 were bred and housed in pathogen free conditions [25, 26]. All mice used in these experiments were 5–12 weeks old. All use of laboratory animals was done with approval of the Animal Care and Use Committee (IACUC protocol #15–801) at Brigham Young University.

### T cell isolation and activation

CD4^+^ T cells were isolated from the spleens of LLO56 and LLO118 TCR transgenic (Tg) mice [26]. Spleen single cell suspensions from LLO56 and LLO118 mice were purified using a negative selection CD4^+^ T cell isolation kit (Miltenyi Biotec) [17]. T cells were isolated from the spleen of LLO56, LLO118, LLO56-CD5KO, and LLO118-CD5KO mice. Spleens were homogenized and passed through a nylon mesh cell strainer. The single-cell suspension was resuspended in R10 medium containing RPMI 1640, 10% of FBS (HyClone), 1% Glutamax (Gibco by Life Technologies), and 0.5% Gentamicin (Life Technologies), then transferred to a 6-well plate (1x10^6^ cell/ml) and loaded with 1 μM of *Listeria monocytogenes* peptide LLO_190-205_. For T cell isolations, mice were euthanized using CO_2_ inhalation.

### Antigen presenting cell isolation

Bone marrow derived macrophages (BMDM) were obtained from B6/C57 mouse femurs and tibias and were cultured at 37°C and 5% CO_2_ and matured for 7 days in macrophage medium with DMEM (HyClone), 10% FBS (HyClone), 20% supernatant from L929 mouse fibroblast as a source of macrophage colony-stimulating factor (M-CSF), 5% heat inactivated horse serum (Sigma), 1 mM Na Pyruvate (Gibco by Life Technologies), 1.5 mM L-glutamine (Thermofisher), 1100X Penicillin/Sreptomycin (Gibco by Life Technologies). Harvested cells were plate in an 8-chamber cover glass where they were loaded with the *Listeria monocytogenes* peptide LLO_190-205_ overnight. For bone marrow derived macrophage isolations, mice were euthanized using CO_2_ inhalation.

### Calcium imaging

Naïve T cells were incubated with 1 μM of Fura-2AM (Invitrogen) for 30 minutes at 37°C and 5% CO_2_ in Ringers imaging solution (150 mM NaCl, 10mM glucose, 5 mM of HEPES, 5 mM of KCl, 1mM MgCl_2_, and 2 mM CaCl_2_, pH 7.4), washed, and then incubated in Ringers solution for another 30 minutes at 37°C. 200,000 Fura-2AM loaded naïve CD4^+^ T cells were pipetted onto 200,000 bone marrow derived macrophages that were previously incubated with 1 μM of *Listeria monocytogenes* peptide LLO_190-205_ overnight. Imaging was performed in Nunc 8-chamber covered glass slides (155411, Thermo Scientific). For day 3 and day 8 stimulation time points, LLO118 or LLO56 splenocytes were incubated overnight with 1 μM of *Listeria monocytogenes* peptide LLO_190-205_. Calcium imaging was performed at room temperature using an Olympus IX51 inverted microscope equipped with a xenon arc lamp. Fura-2AM loaded T cells were excited at 340 nm and 380 nm excitation filters and capture by a florescence microscope camera (Q Imaging Exi Blue) using a 20x objective (N.A. 0.75). Images (340/380/transmitted) were recorded at 3 second intervals over 20 minutes. Each individual cell fluorescence was normalized with the first recorded value according to the equation (F-Fo)/Fo where F is the fluorescence at the specific time point, and Fo is the fluorescence value at time 0 [34].

### Flow cytometry

Calcium mobilization was also measured using flow cytometry and the high affinity calcium indicator Fluo-4 (ex:470–490 nm and em: 520–540 nm). Cells were surface stained with an anti-CD4^+^-APC antibody (17–0041; eBioscience). T cells were loaded for 30 mins as previously published with pluronic acid and 1mM Fluo-4-acetoxymethyl ester (Invitrogen) in Ringer solution (150 mM NaCl, 10 mM glucose, 5 mM of HEPES, 5 mM of KCl, 1 mM MgCl_2_, and 2 mM CaCl_2_, pH 7.4) [35]. Intracellular calcium mobilization was initiated by adding 50 ng/ml of PMA (phorbol 12-myristate 13-acetate) and 1 μg/mml of ionomycin [36]. For further analysis done in FlowJo, the lymphocyte population was gated in a forward and side scatter gate and singlets. From this gate a second gate was created specific for CD4^+^ T cells [37]. Intracellular calcium flux was measured in the CD4^+^ T cell gate using the FlowJo kinetics tool.

For CD5 expression analysis, spleen single cell suspensions from naïve and stimulated (day 3 and day 8) were stained with anti-CD5-PE (12–0051; eBioscience), and anti-CD4-APC (17–0041; eBioscience) and analyzed on the flow cytometer (BD Accurri C6).

### Data analysis

Live cell calcium imaging data was analyzed using CellSens Software from Olympus and the 340:380 ratio calculations were performed on randomly selected cells. The standard deviation of the calcium levels from the regression line was determined using GraphPad Prism. For calcium flow cytometry measurements, FlowJo kinetics tool was used to determine the area under the curve (AUC) [35, 38, 39]. All assays were performed at least three times in triplicate and significant values were determined using student T test in GraphPad Prism.

## Results

### LLO118 and LLO56 T helper cells have different responses to antigen and CD5 expression levels

To examine the role of CD5 in regulating calcium signaling in the primary response of helper T cells, we used LLO56 and LLO118 T cells which are specific for the same epitope of *Listeria monocytogenes* (listeriolysin O, LLO_190-205_) [25]. These LLO118 and LLO56 T cells differ in their *in vivo* responses upon *L*. *monocytogenes* infection; LLO118 helper T cells have a better primary response and LLO56 helper T cells exhibit a better secondary response [25]. Additionally, naïve LLO56 T cells have higher levels of CD5 and produce more IL-2 upon stimulation compared to those from LLO118 T cells [25, 26]. We hypothesized that these differences in CD5 levels and T cell function would allow us to better understand the role of CD5 in calcium signaling and T cell activation in a primary response (See Table 1).

**Table 1:** Summary of differences between LLO56 and LLO118 T cells.

	LLO56	LLO118
**Primary Response**	+	+++
**Secondary Response**	+++	+
**IL-2 Response**	+++	++
**CD5 Expression (naïve T cells)**	+++	+

### LLO56 naïve helper T cells have higher calcium mobilization in vitro

**Fig 1:**
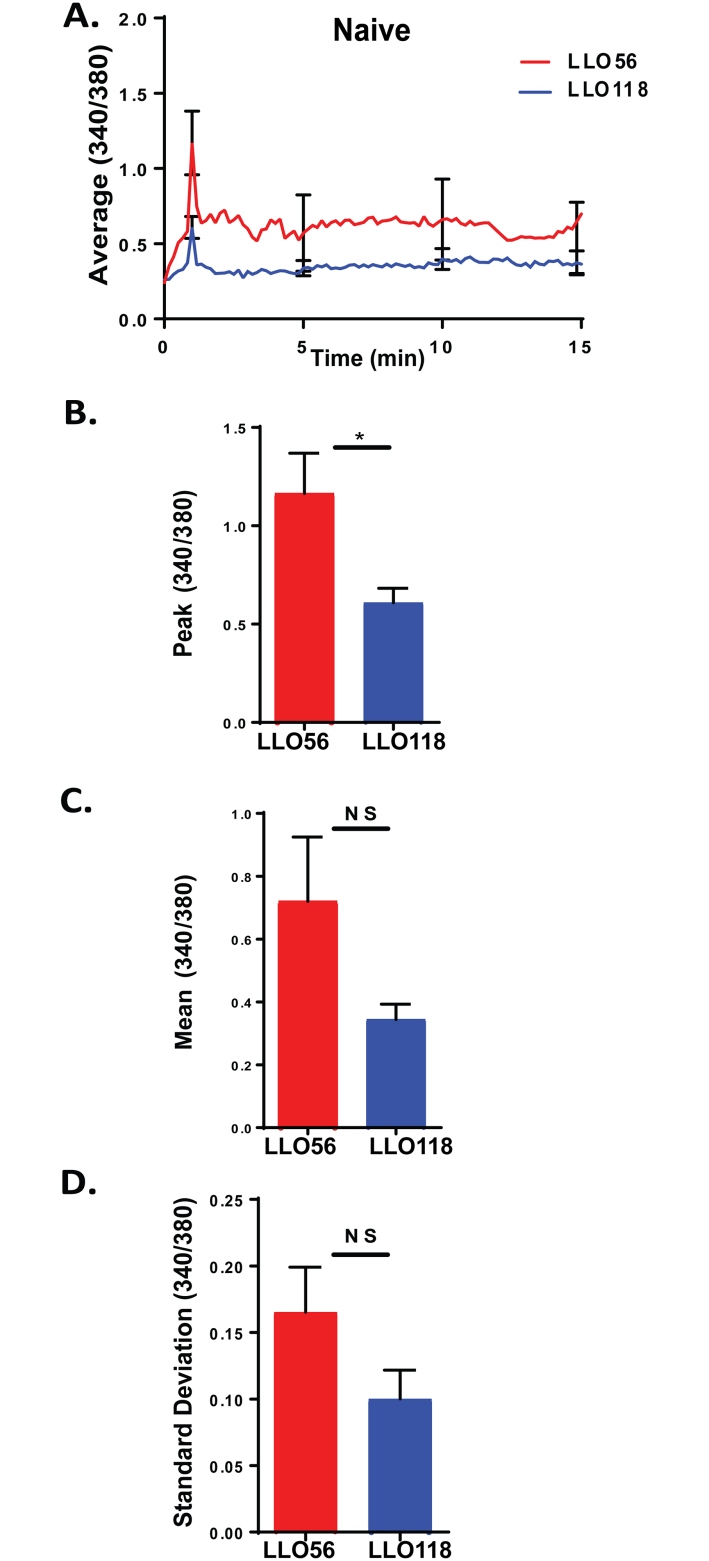
LLO56 naïve helper T cells have higher calcium mobilization *in vitro*. Naïve T cells from LLO56 and LLO118 TCR transgenic mice were obtained from the spleen using negative selection and calcium levels were measured using live cell microscopy. T cells were added to antigen presenting cells (bone marrow derived macrophages) that were loaded overnight with the *L*. *monocytogenes* peptide (LLO_190-205_). **A.** Average curves of intracellular Ca^2+^ mobilization from LLO56 and LLO118 naïve T cells (340/380 ratios) (n = 40^+^). Error bars show the SEM at the influx peak and every 5 minutes after the peak (n = 40^+^). **B.** Statistical analysis of peak calcium influx of stimulated LLO56 and LLO118 naïve T helper cells (n = 40^+^). **C.** Statistical analysis of the sustained intracellular Ca^2+^ levels (Average 340/380 values between minutes 5 and 20) after initial stimulation response (n = 40^+^). **D.** Standard deviation was determined by linear regression analysis and shows variability in the calcium signal for each group (n = 40^+^). (* = p < .05; NS = not significant).

To determine how CD5 plays a role in the primary immune response of LLO56 and LLO118 T cells, we first analyzed the calcium signaling of naïve T cells isolated and purified from the spleens of LLO56 and LLO118 TCR Tg mice. Calcium mobilization was measured using live cell imaging after loading the T cells with Fura-2AM and adding them to 8-chamber slides containing antigen presenting cells loaded overnight with the *L*. *monocytogenes* peptide (LLO_190-205_). A calcium profile was generated by combining measurements (40^+^ cells) from 4 different experiments taking readings every 3 seconds over a 20-minute time span (Fig 1A). Upon stimulation with LLO_190-205_ peptide, LLO56 T helper cells have higher peak calcium influx levels compared to LLO118 T cells (Fig 1B). There are not any significant differences in the mean calcium levels and variability (standard deviation) of the calcium signal between LLO56 and LLO118 T cells (Fig 1C and 1D) [40]. Thus, naïve LLO56 (CD5-high) and naïve LLO118 (CD5-low) T cells have significantly different peak calcium mobilization profiles.

### LLO56 naïve T helper cells have higher levels of CD5 surface expression

**Fig 2:**
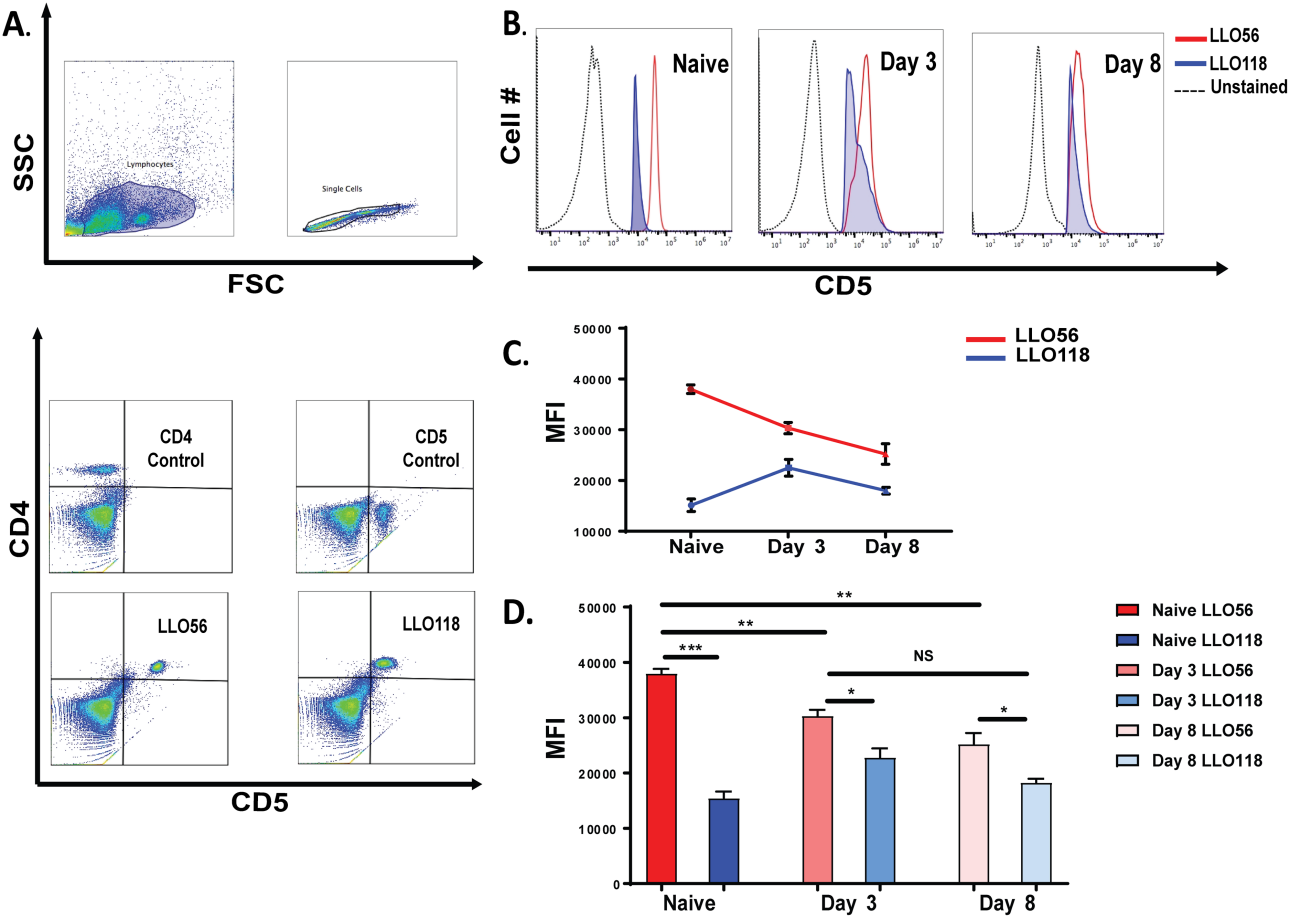
Naïve LLO56 T helper cells have higher levels of CD5. Flow cytometry analysis of CD5 expression of LLO56 and LLO118 T helper cells was done using LLO118 and LLO56 splenocytes at different time points after stimulation with the LLO_190-205_ peptide from *L*. *monocytogene*s. **A.** Gating strategy for measuring CD4 and CD5 mean fluorescent intensity on LLO118 and LLO56 T cells. **B.** CD5 levels of naïve T cells, T cell stimulated for 3 days, and T cells stimulated for 8 days. T helper cells from LLO56 (red line) overlaid with the CD5 levels from LLO118 (shaded blue). Unstained cells are also included (black dots). **C-D.** Comparison of mean fluorescence intensity (MFI) profiles for LLO56 and LLO118 expression levels of CD5 at different time points were determined by flow cytometry quantitative analysis. (* = p < .05; ** = p < .01; *** = p < .001; NS = not significant).

Previous work has shown that naïve LLO56 T cells have higher expression of CD5 compared to naïve LLO118 T cells [25]. We wanted to know what happened to the levels of CD5 at the post-stimulation time points (day 3 and day 8) that we examined in this study. As previously reported, LLO56 naive T helper cells showed higher CD5 expression than naïve LLO118 T helper cells. However, upon stimulation, the CD5 expression differences between LLO56 and LLO118 T cells decrease over the course of 8 days of stimulation (Fig 2A and 2B). To further confirm CD5 expression a mean fluorescent intensity (MFI) profile was done, which confirmed significant CD5 expression levels differences between naïve LLO56 T cells with Day 3 and Day 8, however LLO56 did not have significant differences between Day 3 and Day 8 (Fig 2C and 2D).

### LLO118 T cells have higher peak calcium influx on day 3 post-stimulation

**Fig 3:**
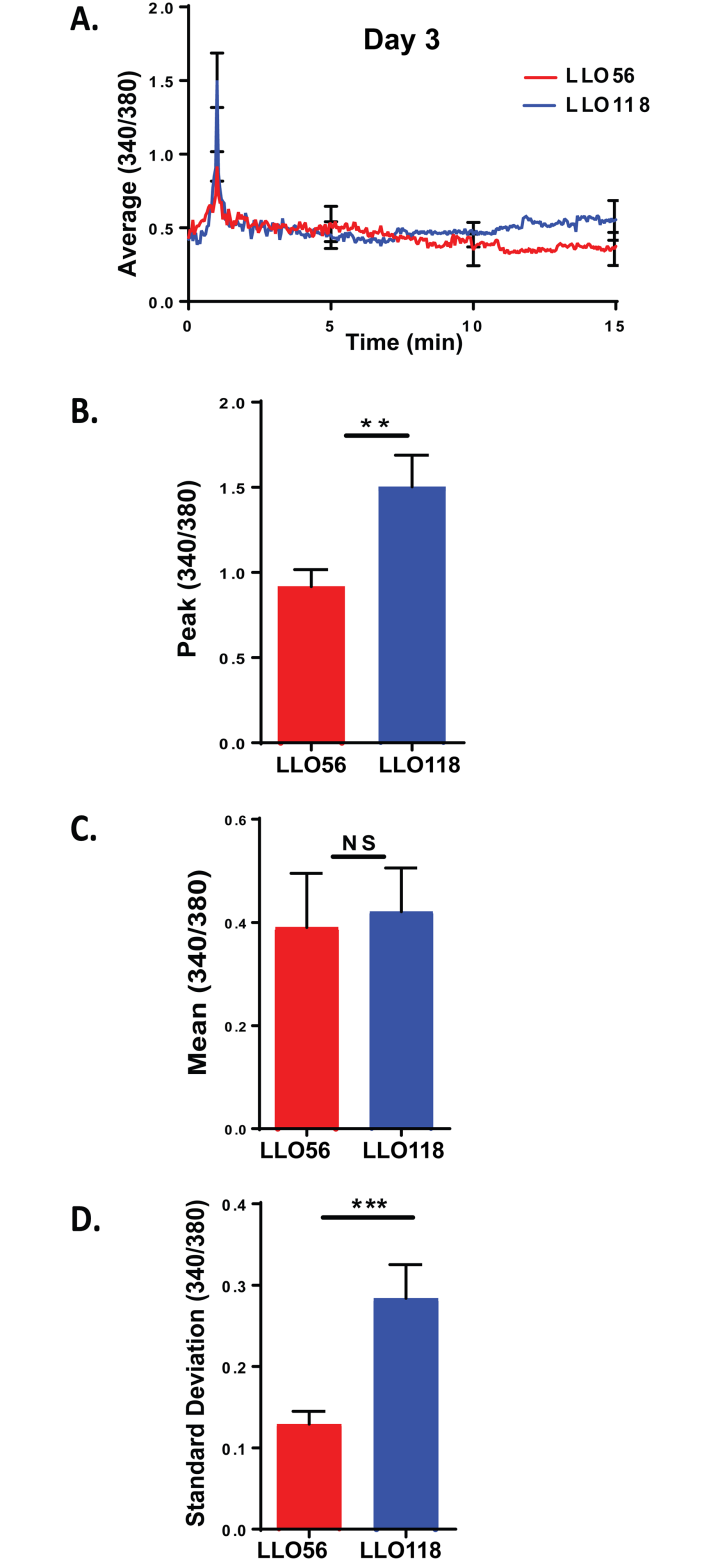
LLO118 helper T cells have higher calcium signaling on day 3 post stimulation. LLO56 and LLO118 splenocytes were isolated and cultured with the LLO_190-205_ peptide from *L*. *monocytogenes* for 72 hours *in vitro*. 24 hours before live imaging, a second set of splenocytes were isolated and cultured in an 8-chamber slide loaded with LLO_190-205_ peptide of *L*. *monocytogenes* for use as antigen presenting cells. T cells were stained with Fura-2AM, added to the antigen presenting cells and Ca^2+^ influx was measured. **A.** Average curves of intracellular Ca^2+^ mobilization from stimulated LLO56 and LLO118 splenocytes (340/380 ratios; n = 30) on day 3 post stimulation. Error bars show the SEM at the influx peak. **B.** Statistical analysis of peak calcium influx of stimulated LLO56 and LLO118 naïve T helper cells (n = 30^+^). **C.** Statistical analysis of the sustained intracellular Ca^2+^ levels (Average 340/380 values between minutes 5 and 20) after initial stimulation response (n = 30^+^). **D.** Standard deviation was determined by linear regression analysis and shows variability in the calcium signal for each group (n = 30^+^). (** = p < .01; *** = p < .001; NS = not significant).

To determine whether the Ca^2+^ influx difference seen in naïve T cells were maintained over the course of a primary response to infection, we measured calcium influx for LLO118 and LLO56 T helper cells 3 days post-stimulation with *L*. *monocytogenes* peptide (LLO_190-205_) (Fig 3A). In contrast to naïve T cells, day 3 post-stimulated LLO118 T cells have significantly higher peak levels of calcium influx than LLO56 T helper cells (Fig 3B). While there were no differences in mean Ca^2+^ levels (Fig 3C), LLO118 did have significantly higher variability in the calcium signal (standard deviation). Thus, on day 3 post-stimulation LLO56 T cells had significantly lower peak calcium influx and lower variability compared to LLO118 T cells.

### LLO56 and LLO118 have similar in vitro calcium responses on day 8 post stimulation

**Fig 4:**
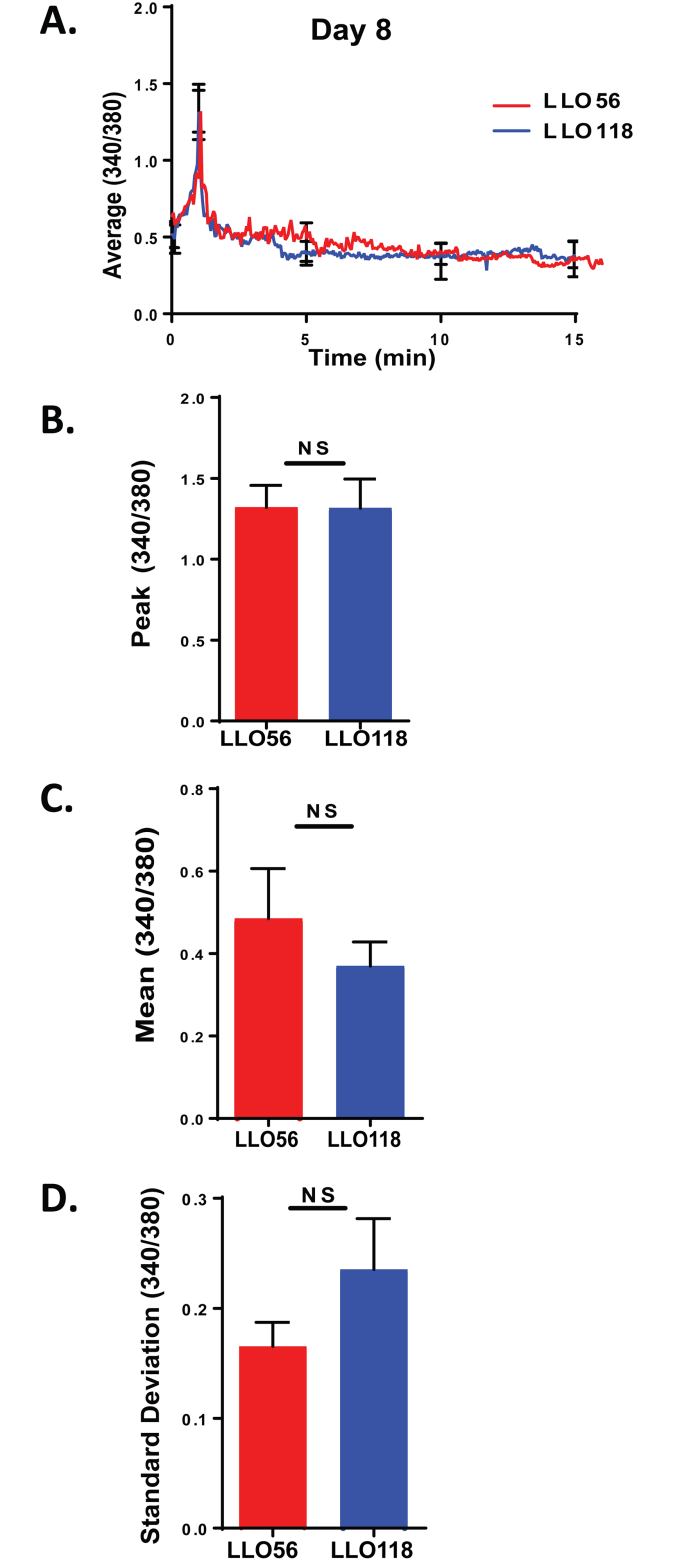
No calcium differences between LLO56 and LLO118 on day 8 post stimulation. LLO56 and LLO118 transgenic splenocytes were isolated and cultured with the LLO_190-205_ peptide for a week. 24 hours before live imaging, a second set of splenocytes were isolated and cultured in an 8-chamber slide loaded with LLO_190-205_ peptide of *L*. *monocytogenes* for use as antigen presenting cells. 8 days stimulated T cells were stained with Fura-2AM and Ca^2+^ influx was measured using live imaging microscopy. **A.** Average curves of intracellular Ca^2+^ mobilization from stimulated LLO56 and LLO118 splenocytes (340/380 ratios; n = 30) on day 8 post stimulation. Error bars show the SEM at the influx peak. **B.** Statistical analysis of peak calcium influx of stimulated LLO56 and LLO118 naïve T helper cells (n = 30^+^). **C.** Statistical analysis of the sustained intracellular Ca^2+^ levels (Average 340/380 values between minutes 5 and 20) after initial stimulation response (n = 30^+^). **D.** Standard deviation was determined by linear regression analysis and shows variability in the calcium signal for each group (n = 30^+^). (NS = not significant).

To further characterize the LLO118 and LLO56 Ca^2+^ response, we isolated splenocytes and co-cultured them with the *L*. *monocytogenes* peptide for 8 days. On day 8 post-stimulation, the average Ca^2+^ profiles were similar to each other (Fig 4A). Upon evaluation, there were no significant differences between LLO118 and LLO56 T cells in calcium peak, mean, or standard deviation on day 8 post-stimulation (Fig 4B–4D).

### Calcium flow cytometry data correlates with calcium microscopy data

**Fig 5:**
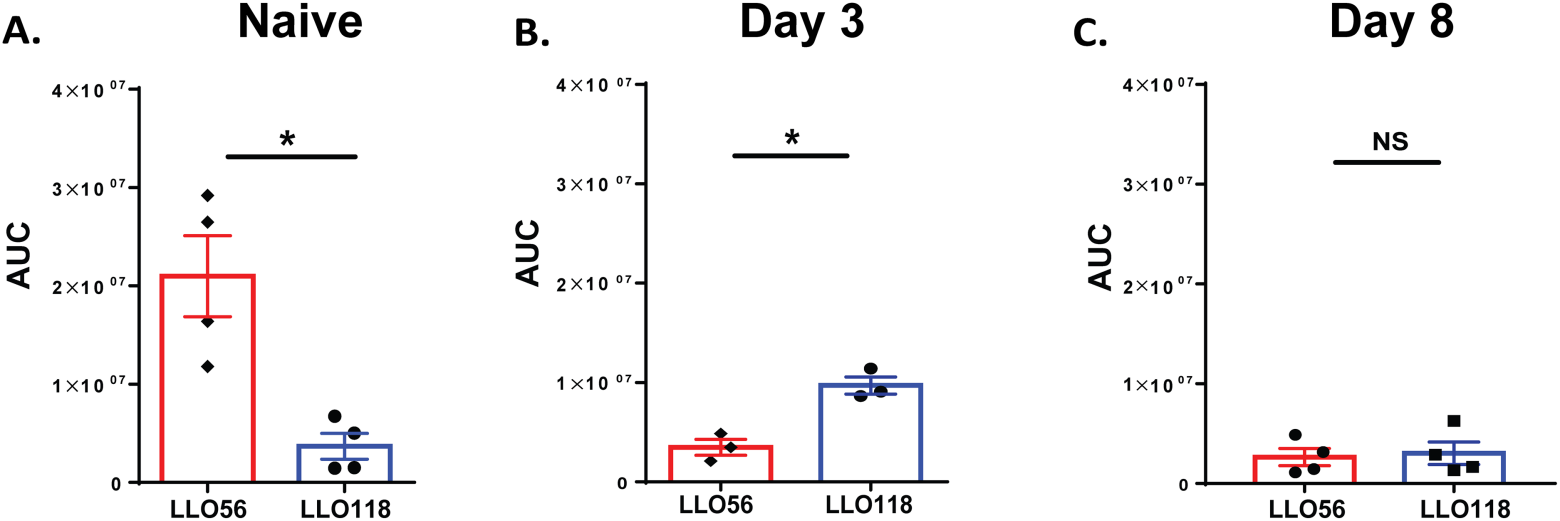
Flow cytometry calcium analysis confirms improved calcium mobilization for naïve LLO56 T cells and higher calcium mobilization for LLO118 T cells at day 3 post-stimulation. LLO56 and LLO118 splenocytes were isolated and cultured at different time points with the LLO_190-205_ peptide. Calcium levels were quantified using the FlowJo kinetics tool to determine the area under the curve (AUC) for each sample. Calcium mobilization levels for LLO118 and LLO56 are quantified (mean ± SEM of the area under the curve). **A.** Statistical analysis of naïve LLO118 and LLO56 T helper cell calcium mobilization after activation with PMA and Ionomycin. **B.** Statistical analysis of day 3 post stimulated LLO118 and LLO56 calcium mobilization and **C.** Statistical analysis of day 8 post stimulated LLO118 and LLO56 calcium mobilization. (NS = not significant).

To confirm the results obtained in live cell calcium microscopy and evaluate the role of TCR independent calcium signaling, we isolated LLO56 and LLO118 T cells and measured Ca^2+^ mobilization using flow cytometry. Cells were labeled with Flou-4AM and stimulated with PMA and ionomycin. Flou-4AM fluorescence was examined before and after stimulation. Measurements were collected for naïve T helper cells, day 3, and day 8 post-stimulated T cells. The data was consistent with our previous live cell imaging findings in which naïve LLO56 T helper cells and day 3 post-stimulated LLO118 T cells had higher Ca^2+^ mobilization compared to their counterparts while no calcium mobilization differences were seen at day 8 between LLO118 and LLO56 T cells (Fig 5A–5C and Table 2). Collectively, these data show CD5 expression levels and calcium signaling changes over the course of a primary response in LLO118 and LLO56 T cells (Table 2, Figs 2 and 5). Our live cell microscopy calcium imaging and flow cytometry calcium analysis differ in the parameters measured and the stimulation used (cells were stimulated in a TCR-dependent manner for live cell calcium imaging and in a TCR-independent manner for flow cytometry analysis). This calcium data is consistent with the TCR independent cytokine production differences between LLO118 and LLO56 identified by Persaud et al, in which they demonstrated that the LLO118 and LLO56 naive T cell response was set during thymic selection. Naive LLO56 T cells have higher expression of CD5, suggesting increased affinity for self-peptide, and produce higher levels of IL-2 even when stimulated in a TCR independent manner [26].

**Table 2:** Summary of CD5 and calcium findings for LLO56 and LLO118.

	LLO56		LLO118	
	CD5 Expression	Ca^2+^ influx	CD5 Expression	Ca^2+^ mobilization
**Naïve T cell**	**+++**	**+++**	**+**	**+**
**Day 3**	**++**	**++**	**+**	**+++**
**Day 8**	**++**	**++**	**+**	**++**

### CD5 expression in naïve LLO56 T helper cells is correlated with higher Ca^2+^ mobilization

**Fig 6:**
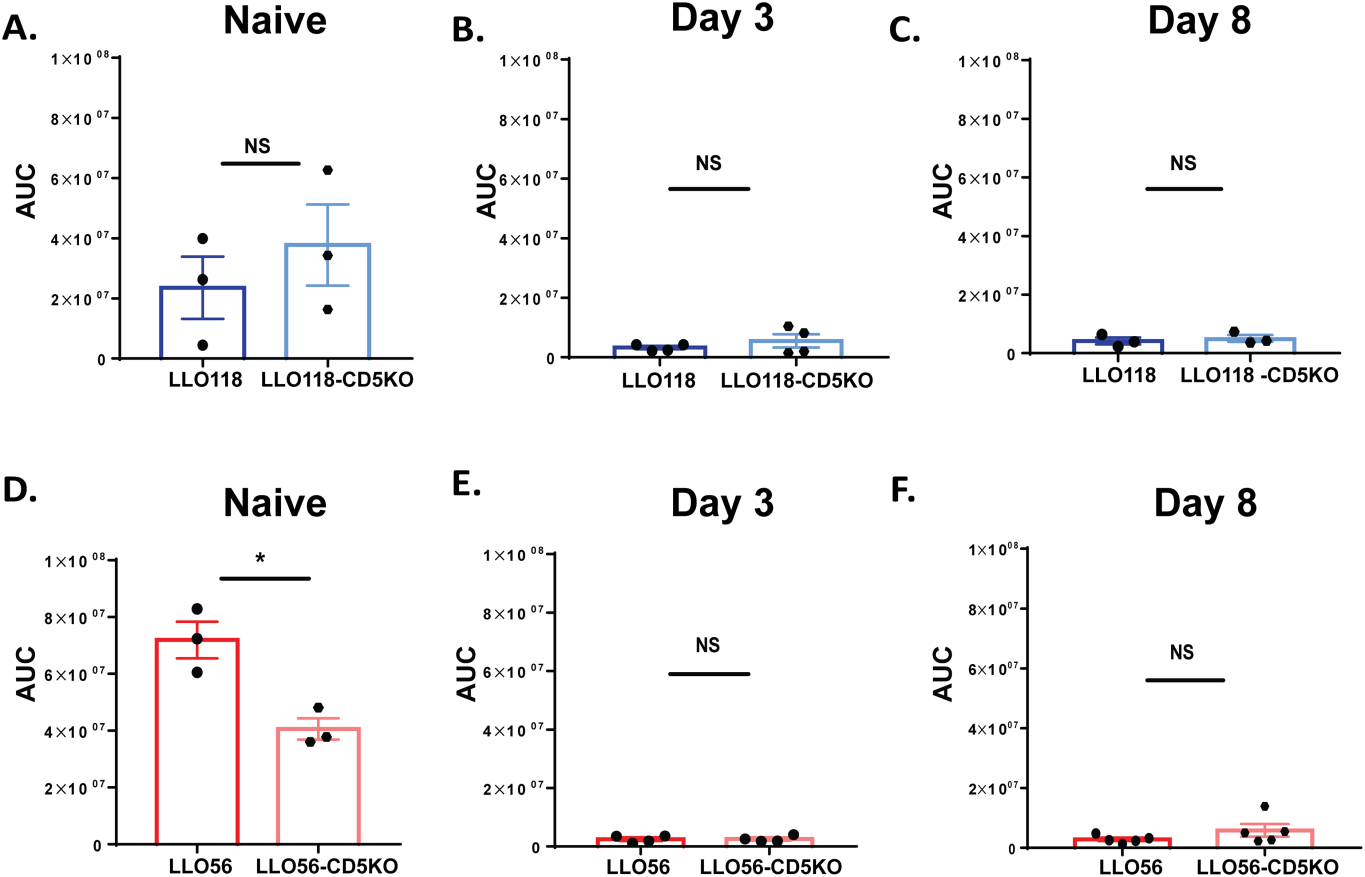
CD5 expression in naïve LLO56 T helper cells is correlated with higher Ca^2+^ mobilization. Flow cytometry analysis was performed to determine Ca^2+^ mobilization levels in LLO56, LLO118, LLO56-CD5 knockout and LLO118-CD5 knockout T cells stimulated with the *L*. *monocytogenes* peptide (naïve, day 3, and day 8 time points). Calcium levels were quantified using the FlowJo kinetics tool to determine the area under the curve (AUC) for each sample (mean ± SEM). **A-C.** Statistical analysis of calcium mobilization of LLO118 and LLO118-CD5 knockout T cells stimulated with PMA/Ionomycin. Data is shown for naive (**A**), day 3 post stimulation (**B**) and day 8 post stimulation (**C**). **D-F.** Statistical analysis of calcium mobilization of naïve LLO56 and LLO56-CD5 knockout T cells stimulated with PMA/Ionomycin. Data is shown for naive (**D**), day 3 post stimulation (**E**) and day 8 post stimulation (**F**). (* = p < .05; NS = not significant).

To further investigate the role CD5 expression plays in Ca^2+^ mobilization, we measured the calcium signal in T cells from LLO118-CD5 knockout and LLO56-CD5 knockout mice. We found in the LLO118 T cells (CD5-low) that calcium mobilization was not significantly different from LLO118-CD5 knockout T cells at any of the three time points (Fig 6A–6C). Conversely, naïve LLO56-CD5 knockout T helper cells had significantly lower calcium levels compared to the naïve LLO56 T cells (CD5-high) (Fig 6D). There was no calcium mobilization difference between LLO56 and LLO56-CD5 knockout T cells at day 3 or day 8 post-stimulation (Fig 6E and 6F). Thus, in naïve LLO118 T cells (CD5-low), CD5 does not appear to play a strong role in regulating calcium mobilization at any of the time points. However, CD5 expression is important in regulating calcium mobilization in the naïve LLO56 T cells (CD5-high) during the initial response to antigen, but as CD5 levels decrease over time, its role in regulating calcium also decreases.

## Discussion

In this study, we examined the role of CD5 in regulating T cell activation during a primary response using two T helper cells, LLO56 and LLO118, which bind to the same *L*. *monocytogenes* epitope and have different levels of CD5 on the surface upon completion of thymic development [25, 26]. Because of the described negative regulatory role of CD5 in the thymus and the prevalence of CD5-high cells in memory cells, we wondered how CD5 influences T cell immune response at a post-thymic level. We found significantly different Ca^2+^ signaling levels between LLO56 and LLO118 T helper cells at the naïve and day 3 time points. The distinct Ca^2+^ mobilization patterns of LLO56 and LLO118 likely influence their particular responses to antigen, similar to observations made in B cells in which unique Ca^2+^ mobilization controls distinct B cell activation phenotypes [20, 41]. Previous work has defined the important role of CD5 during T cell thymic development and that CD5-high cells are enriched in memory T cell populations, but how CD5 functions during the primary response stage of T helper cells has not been well defined. Here we characterized the role of CD5 expression and calcium mobilization in these CD5-high and CD5-low T cells over the course of 8 days. We found that naïve LLO56 T helper cells (CD5-high) have significantly higher calcium mobilization compared to the LLO56-CD5 knockout T cells, but at later time points the removal of CD5 did not significantly alter LLO56 calcium mobilization. Naïve LLO118 T helper cells (CD5-low) exhibit no differences in Ca^2+^ mobilization relative to their CD5 knockout counterpart. Thus, we found naive CD5-high T cells have improved calcium mobilization to an antigen they have never seen before.

T cell development shapes the T cell population by removing strongly self-reactive cells and helping determine future immune responses. T cells that are moderately self-reactive may be able to pass positive selection and evade negative selection and circulate in the periphery. These self-reactive cells, marked by high levels of CD5, appear to be primed to be the best responders to foreign antigens [26]. CD5 is a known negative regulator of TCR signaling during thymocyte development and its expression is correlated to the relationship of TCR avidity for self-pMHC [25, 26, 28, 42]. Analysis in thymocytes showed that LLO56 and LLO118 CD5 knockout T cells had increased p-ERK and IL-2 production, providing additional evidence that CD5 has a negative regulatory effect in developing thymocytes [26]. However, additional work has demonstrated that CD5-high and CD5-low T cells respond differently to self and foreign antigens, suggesting that CD5 has an important role in thymocyte selection and peripheral T cell function and fate [28, 29].

Recent studies suggest that developing T cell CD5 levels affect naïve T cell responses to foreign antigens in the periphery [15, 43]. While the negative regulatory function of CD5 in the thymus does not appear to be dependent upon engagement with a ligand, the positive co-stimulatory effect of CD5 in the periphery is likely due to CD5 engagement of a ligand (CD5 or CD5L) [30–33]. As previously reported, anti-CD5 antibodies enhance TCR-mediated activation and proliferation in peripheral T cells [29, 44, 45]. This calcium difference observed in naïve T cells is supportive of the previously published finding that LLO56 T cells have significantly higher phosphorylation levels of pERK and production of IL-2 before exposure to antigen, suggesting a role for self-peptide affinity in altering CD5 levels and naive T cell responses. CD5-high T cells respond strongly upon stimulation *in vitro* and have increased IL-2 secretion and greater Erk phosphorylation compared to CD5-low T cells [26]. Since CD5 expression is set by self-peptide reactivity in the thymus, our finding that naïve CD5-high LLO56 T cells have higher calcium influx is consistent with other studies that have shown that increased reactivity to self-peptides results in T cells with improved reactivity to foreign antigens [14, 28, 46].

We found that CD5 expression plays an important role in intracellular Ca^2+^ mobilization for naïve LLO56 helper T cells (CD5-high). CD5-high T cells have stronger avidity for self-peptide. It has been suggested that the enhanced activation response to foreign pathogens of CD5-high T cells could be due to their ability to more efficiently use self-peptide as a co-agonist peptide in the periphery [15]. Additionally, CD5-high cells have better basal TCR signaling and improved functional characteristics which correlate with better response to foreign peptide [28]. Studies in naïve cytotoxic T cells suggest that the gene expression profile of CD5-high T cells transcriptionally engage into proliferative and effector functions faster than CD5-low T cells [46]. Furthermore, CD5 appears to help with CD5-high naïve T cell survival after antigen recognition [47]. In fact, T cells with high CD5 levels may maintain diversity within the memory population, which may outweigh the cost of increased self-reactivity [15]. Additionally, T cells with high CD5 expression are enriched in memory cell populations, suggesting that when designing vaccines, CD5 levels and self-peptide and foreign peptide interactions are an important consideration [14, 15].

The data presented here helps to elucidate the role that CD5 plays in regulating calcium signaling in naïve cells early after cell activation during an *in vitro* primary response. We plan to further investigate whether the unique Ca^2+^ profiles of LLO56 and LLO118 T cells are consistent in an *in vivo* model and further quantify the role CD5 plays in effector and memory T cells. These future studies will help elucidate how CD5 influences naïve T cell responses and its potential role in memory T cell generation and maintenance.
